# Cocktail of carbohydrases from *Aspergillus niger*: an economical and eco-friendly option for biofilm clearance from biopolymer surfaces

**DOI:** 10.1186/s13568-021-01183-y

**Published:** 2021-02-04

**Authors:** Arashdeep Kaur, Sanjeev Kumar Soni, Shania Vij, Praveen Rishi

**Affiliations:** Department of Microbiology, Panjab University, Sector-25, Chandigarh, 160014 India

**Keywords:** Antibiotics, Biofilms, Carbohydrases, Dispersal, Medical devices, Response surface methodology (RSM)

## Abstract

Biofilm formation on both biotic and abiotic surfaces accounts for a major factor in spread of antimicrobial resistance. Due to their ubiquitous nature, biofilms are of great concern for environment as well as human health. In the present study, an integrated process for the co-production of a cocktail of carbohydrases from a natural variant of *Aspergillus niger* was designed. The enzyme cocktail was found to have a noteworthy potential to eradicate/disperse the biofilms of selected pathogens. For application of enzymes as an antibiofilm agent, the enzyme productivities were enhanced by statistical modelling using response surface methodology (RSM). The antibiofilm potential of the enzyme cocktail was studied in terms of (i) in vitro cell dispersal assay (ii) release of reducing sugars from the biofilm polysaccharides (iii) the effect of enzyme treatment on biofilm cells and architecture by confocal laser scanning microscopy (CLSM). Potential of the enzyme cocktail to disrupt/disperse the biofilm of selected pathogens from biopolymer surfaces was also assessed by field emission scanning electron microscopy (FESEM) analysis. Further, their usage in conjunction with antibiotics was assessed and it was inferred from the results that the use of enzyme cocktail augmented the efficacy of the antibiotics. The study thus provides promising insights into the prospect of using multiple carbohydrases for management of heterogeneous biofilms formed in natural and clinical settings.

## Key points

- Co-production of carbohydrases from *A. niger*, a soil isolate, optimized by RSM.
- Validation of biofilm dispersal/clearence from coverslips and biopolymer surfaces.
- Enzyme cocktail potentiated the efficacy of antibiotics.

## Introduction

Biofilms, also referred to as “city of microbes”, are a consortium of microbial cells encased in extracellular polymeric substance (EPS). EPS is responsible for the stability and recalcitrance of biofilm cells to host defences as well as against environmental extremities (Del Pozo and Patel 2007). The protective environment provided by the biofilm architecture makes cells much more resistant to different antimicrobials. Up to 1000-fold increased antibiotic tolerance has been reported in biofilm cells as compared to their planktonic counterparts (Rogers et al. 2010). Moreover, higher doses of antibiotics for longer periods are needed to combat biofilms which can induce antibiotic resistance in pathogens. The reasons for enhanced antimicrobial resistance include physical impedance, enzymatic inactivation of the drugs, and complex metabolic layering in biofilm-associated cells (Vasudevan et al. 2019). Due to their resistant nature, the biofilms which are ubiquitous and are prevalent in natural, hospital, and industrial settings, pose a serious public health concern. In addition to the therapeutic challenge, biofilms lead to severe infections in patients with indwelling medical devices. Increased propensity of biofilm formation has been observed on medical devices and the same has been implicated in serious medical device related infections (Stewart and Bjarnsholt 2020).

Traditionally, various physical and chemical methods are employed for the control and removal of biofilms. Chemicals such as surfactants, chelating agents, hypochlorite, monochloramine, and concentrated urea are employed for control of biofilms. However, the use of chemicals is non-ecofriendly, expensive and also leads to corrosion of surfaces. Looking at these challenges posed by biofilms, it becomes imperative to combat biofilms by alternate and more environment friendly means. Till date, various strategies have been investigated for the management of biofilms and one of them is to disperse the biofilms by targeting different components of EPS. The EPS consists of different biopolymers such as carbohydrates, proteins, glycopeptides, nucleic acids, lipids, glycolipids and levan (Hobley et al. 2015; Karygianni et al. 2020). Out of these biopolymers, carbohydrates are the most studied components of biofilm and can be present as homo-polysaccharides or as hetero-polysaccharides (Koo et al. 2017; Sharahi et al. 2019). EPS provides many important functions for the establishment and persistence of biofilms including structural stability, physical and chemical defence against antimicrobials and the host immune system, adhesion and aggregation of microbial cells, desiccation tolerance, sorption of organic and inorganic compounds and can act as a carbon source in times of nutrient starvation (Flemming and Wingender 2010; Limoli et al. 2015; Watters et al. 2016). Due to their importance in establishing and maintaining the biofilm architecture, the interest of the scientific community has now been diverted towards targeting these biofilm EPS.

Various agents including enzymes, antibiofilm peptides, and dispersal molecules that target the biofilm architecture have been found to be promising as anti-biofilm agents (Fleming and Rumbaugh 2017). Recently, we reported the use of an enzyme consortium obtained from *Aspergillus niger*, a soil isolate, for the clearance of biofouling and black gunk developed on the surface of kitchen drainage pipes (Kaur et al. 2020a). In continuation, in the present study, yet another application of the same enzyme consortium i.e. eradication of the biofilms from biomedical surfaces was investigated. Before using enzyme cocktail for biofilm removal, the enzyme production from *A. niger* was optimized by statistical modelling using response surface methodology (RSM) to achieve maximum enzyme productivities. Further, the enzymes were characterized and the potential of these enzymes in combination with antibiotics was also explored. The dual functionality of this combination i.e. dispersal of biofilm EPS by enzyme cocktail coupled with the antibacterial activity of antibiotics might prove to be helpful in reducing the effective concentration of antibiotics, thereby minimising the chances of emergence of antimicrobial resistance due to the multi-hit approach of the two agents. The study envisages a simple approach for the co-production of multiple carbohydrases from a single organism which eliminates the need for procurement of individual enzymes from various sources to act on heterogeneous biofilms thereby proving useful for multiple applications in natural and clinical settings.

## Materials and methods

### Bacterial strains

Bacterial strains of *Staphylococcus aureus* (ATCC 9144), *Escherichia coli* (MTCC 3222) and *Pseudomonas aeruginosa* (MTCC 1934) were used for biofilm production. These strains were maintained on nutrient agar slants and were also preserved as glycerol stocks at − 80 °C.

### Enzyme cocktail

The in-house produced enzyme cocktail (Kaur et al. 2020a) was obtained from cell free supernatant of *A. niger* APS, isolated from soil, (GenBank accession number MN559364; deposited in MTCC with accession number 12975), comprised of cellulases, hemicellulases, pectinase, amylases, and alginate lyase. The enzyme preparation was filter-sterilized using 0.22 µm syringe filters before use.

### Antibiotics

The antibiotics, ampicillin, ciprofloxacin, and chloramphenicol were obtained from Sigma Aldrich, MO, USA. Antibiotics were stored as per manufacturer’s recommendations.

### Conditions for the co-production of the multiple carbohydrases from *A. niger* APS

Various cultural and environmental conditions for the co-production of carbohydrases from *A. niger* APS have been optimized by us. In the present study, RSM was used to identify the optimal values for most significant variables including corn steep liquor, CaSO_4_, MgSO_4_ and tween-80. All other factors which showed positive responses in earlier optimization studies including wheat bran (1.70%), kitchen waste (1.88%), CMC (0.56%), de-oiled rice bran (0.70%), (peptone, 0.76%), (KH_2_PO_4_, 0.02%) and (FeSO_4_, 0.02%) were kept constant in the medium. Surface fermentation was carried out in 250 ml Erlenmeyer flasks. Each flask contained 50 ml medium and inoculation was done with 4 fungal discs cut from the periphery of actively growing cultures of *A. niger* APS grown on PDA plates. Incubation was done at 30 °C in stationary state for 6 days. The enzyme cocktail was obtained from the surface cultures of *A. niger*. After incubation period, the contents of the flasks were centrifuged at 10,000 rpm at 4 °C for 15 min and the supernatant so obtained was used for enzyme assays.

### Statistical modelling by response surface methodology (RSM) for designing medium, augmenting the co-production of all the carbohydrases

The central composite design of RSM was created to develop a second-order model. The design consisted of 30 experimental runs (11 centre points), with four variables: corn steep liquor (A), CaSO_4_ (B), MgSO_4_ (C) and Tween 80 (D). The factors were examined at 5 levels as very high (+ 2), high (+ 1), centre (0), low (− 1), and very low (− 2) as shown in Table 1. Other components of the medium i.e. wheat bran, kitchen waste, CMC, de-oiled rice bran, peptone, KH_2_PO_4_ and FeSO_4_ were kept constant.

**Table 1:** Randomized central composite design of response surface methodology for evaluating interactions between some important factors influencing enzyme(s) production from *A. niger* APS under surface fermentation

Run	Block	CSL % (A)	CaSO_4_% (B)	MgSO_4_% (C)	Tween 80% (D)
1	Block 1	− 1	− 1	+ 1	− 1
2	Block 1	+ 1	+ 1	− 1	+ 1
3	Block 1	− 1	+ 1	− 1	− 1
4	Block 1	0	0	0	0
5	Block 1	+ 1	− 1	− 1	− 1
6	Block 1	0	0	0	0
7	Block 1	− 1	+ 1	+ 1	+ 1
8	Block 1	+ 1	+ 1	+ 1	− 1
9	Block 1	− 1	− 1	− 1	+ 1
10	Block 1	+ 1	− 1	+ 1	+ 1
11	Block 2	− 1	− 1	+ 1	+ 1
12	Block 2	+ 1	− 1	+ 1	− 1
13	Block 2	+ 1	+ 1	− 1	− 1
14	Block 2	0	0	0	0
15	Block 2	− 1	+ 1	+ 1	− 1
16	Block 2	− 1	− 1	− 1	− 1
17	Block 2	+ 1	+ 1	+ 1	+ 1
18	Block 2	− 1	+ 1	− 1	+ 1
19	Block 2	0	0	0	0
20	Block 2	+ 1	− 1	− 1	+ 1
21	Block 3	0	0	0	0
22	Block 3	0	0	0	− 2
23	Block 3	0	0	+ 2	0
24	Block 3	0	0	− 2	0
25	Block 3	0	0	0	0
26	Block 3	0	+ 2	0	0
27	Block 3	+ 2	0	0	0
28	Block 3	0	0	0	+ 2
29	Block 3	0	− 2	0	0
30	Block 3	− 2	0	0	0

For CSL; − 2 = 0.4%, − 1 = 0.6%, 0 = 0.8%, + 1 = 1.0%, + 2 = 1.2%; CaSO_4_; − 2 = 0.01%, − 1 = 0.015%, 0 = 0.02%, + 1 = 0.025, + 2 = 0.03%; MgSO_4_; − 2 = 0.01%, − 1 = 0.015%, 0 = 0.02%, + 1 = 0.025, + 2 = 0.03%; Tween 80; − 2 = 0.02%, − 1 = 0.04%, 0 = 0.06%, + 1 = 0.08%, + 2 = 0.1%

A second-order model was selected for predicting optimal points and was expressed as:

$${\text{Y}} = {\text{bo}} + \sum {{\text{bi}}\;{\text{xi}}} + \sum {\sum {{\text{bij}}\;{\text{xi}}\;{\text{xj}}} } + \sum {{\text{bii}}\;{\text{x}}^{2} {\text{i}} + {\text{e}}} .$$

where Y is the measured response; bo, bi, bij, bii are constant, and regression coefficients of model; xi and xj are levels (codes values) of independent variables; e is random error.

The resulting model was analyzed using ‘analysis of variance’ (ANOVA), p, and F values as well as the values of regression coefficients. Contour plots were also obtained by using Design-Expert software version 7.0 to illustrate the relationship between the variables. Accuracy and general ability of the polynomial model were evaluated by the determination coefficient (R^2^).

### Enzyme assays

Cellulase (CMCase, FPase and β-1,4-glucosidase), hemicellulase (xylanase and mannanase), pectinase and glucoamylase activities in cell free supernatant were assayed by quantification of reducing sugars liberated by these enzymes from respective substrates followed by estimation using the 3,5-dinitrosalicylic acid (DNS) method (Miller 1959). The activities of enzymes were expressed in International Units (IU) where one unit each of the CMCase, FPase, β-glucosidase, xylanase, mannanase, pectinase and glucoamylase is equivalent to the enzyme that releases one µmole of end product in one min under standard assay conditions. α-Amylase assay was performed by the method of Fuwa 1954 wherein one IU is equivalent to the amount of enzyme which reduces the color of starch-iodine complex by 10% in 10 min. In case of alginate lyase, one unit has been defined as an increase in optical density (235 nm) of 0.010 per min (Sawabe et al. 1992).

### Characterization of individual enzyme present in the cocktail obtained from *A. niger* APS

The enzyme mixture comprising multiple carbohydrases was characterized by studying the following parameters. The experiments were performed in duplicates and the observations were made by taking the average of the two readings.

#### Temperature activity profiles

The temperature optima for all the enzymes present in the cocktail was determined by assaying respective activities at different temperatures range of 25 °C, 40 °C, 50 °C, 60 °C, 70 °C, 80 °C, 90 °C and 100 °C at pH 4.0 in acetate buffer (0.1 M).

#### pH activity profiles

Optimum pH for various enzyme was determined by assaying respective activities at 50 °C using respective substrate solutions made in different buffer systems with pH ranging from 3.0 to 10.0.

#### Thermostability profiles

The thermostability profiles of all the individual carbohydrases was studied by incubating the enzyme preparations, separately in 0.1 M acetate buffer, pH 4.0 at 50 °C and 60 °C for 168 h. The samples were withdrawn after every 24 h and the residual activity was determined by the standard assay under normal conditions and expressed in terms of % of control.

#### pH stability profiles

The pH stability profiles of enzymes were studied by incubating the enzyme preparations, separately in buffers at pH 4.0 and pH 5.0 at room temperature. The samples were withdrawn after every 24 h intervals for a period of 10 days and the residual activities were determined by the standard assay under normal conditions and expressed in terms of % of control.

#### Effect of metal salts, EDTA and surfactants on enzyme activities

This was studied by supplementing various metal salts including NaCl, CaCl_2_, MgSO_4_, MnSO_4,_ FeSO_4_, ZnSO_4_, KCl, CuSO_4_, EDTA, Tween 80 and SDS (at a final concentration of 5 mM), separately, in the reaction mixtures and determining the relative enzyme activities under normal assay conditions and expressed in terms of % of control.

### Composition assessment of the enzyme cocktail

The enzyme cocktail obtained from *A. niger* APS was checked for the presence of the other enzymes viz. protease and lipase.

#### Protease assay

The protease activity was initially checked by a well diffusion assay using skimmed milk (1.0%) agar plates. Wells were punched in the skimmed milk agar plates. 150 µl of enzyme supernatant (obtained from *A*. *niger* APS) was added to wells. Positive controls, including proteases (chymotrypsin, trypsin and proteinase K at a concentration of 1.0 mg/ml each) were also run in parallel, in separate wells. Neutralized enzyme cocktail (pH adjusted to 7.0 with 1 N NaOH) was also run to study the effect of acidic pH of the enzyme cocktail, if any. Plates were incubated overnight and were checked for the zone of hydrolysis around the wells.

After performing well diffusion assay, protease activity was also checked, spectrophotometrically, by the method of Li et al. (1997), using casein (1.0%) as substrate. One IU of protease is the amount of enzyme that produces one µmole of tyrosine per minute under standard assay conditions.

#### Lipase assay

Lipase activity was also checked by well diffusion assay using glycerol tributyrate agar plates. For this, wells punched in glycerol tributyrate agar plates were filled with 150 µl enzyme supernatant (obtained from *A. niger* APS). Positive control i.e. different concentrations of commercial lipase (2.0 mg/ml, 1.0 mg/ml, 0.5 mg/ml, 0.25 mg/ml and 0.125 mg/ml) were run in parallel. Plates were incubated and were checked for the zone of hydrolysis around the wells for lipase activity. Further, lipase activity was also checked by autoanalyzer.

#### Biofilm formation on a microtitre plate

The biofilms were developed in sterile polystyrene flat-bottomed microtitre plates by the method described by Stepanović et al. (2000) and reported by us earlier (Kaur et al. 2020a). Briefly, the wells of a sterile polystyrene microplate were filled with 230 μl of medium (Luria broth for *E. coli* and *P. aeruginosa* biofilms and Tryptic soy broth for the development *S. aureus*). Thereafter, 20 μl of overnight grown culture (cell count adjusted to 10^8^ CFU/ml) of the selected pathogens was added into separate each wells. The microtitre plates were incubated at 37 °C. Incubation was done for 72 h in case of *E. coli* and *S. aureus* and 120 h in case of *P. aeruginosa* biofilms. The negative control wells contained broth only. After incubation, the contents of the plate were poured off and washed three times with 250 μl of phosphate buffer saline to remove the planktonic cells (PBS; 0.01 M, pH 7.0). After washing, the biofilms were fixed with 250 μl of ethanol (per well) for 15 min after which the microplates were emptied and air-dried. Thereafter, microplates were stained with 250 μl per well of crystal violet (0.1%) for 10 min. Excess stain was rinsed off with sterile distilled water and the plates were air-dried. The dye bound to the adherent cells was resolubilized with 250 μl of 33% (v/v) glacial acetic acid per well and optical densities were read at 595 nm in a microplate reader.

### In-vitro cell dispersal

The in-vitro cell dispersal assay was performed by the method of Fleming et al. (2017). For the assay, biofilms of the selected parhogens were developed in 24-well non-treated tissue culture plate (Falcon). After incubation, the wells were gently washed with PBS to remove the planktonic cells. Following washing, the biofilms were treated with the enzyme cocktail for 30 min. Controls consisting of PBS and heat-inactivated enzyme cocktail were run in parallel. After treatment, the supernatant was removed from the wells and centrifuged at 10,000 rpm for 10 min in order to pellet the cells. Cell pellets so obtained were resuspended in PBS and were used for CFU quantification. Biofilm remaining on the wells were dispersed carefully using cell scrapper. The scrapped cells were suspended in PBS (0.01 M, pH 7.0) for CFU enumeration. Percent bacterial cell dispersal was calculated by finding the quotient of the total CFU (biofilm-associated plus planktonic) count divided by the planktonic CFU (in the supernatant) count as described by Fleming et al. (2017).

### Degradation of EPS

The EPS from the pathogens was extracted as per Nithya et al. (2010) and the amount of reducing sugar released was determined. Briefly, the biofilms were developed on coverslips. For *E. coli* and *P. aeruginosa* biofilms Luria broth was used and for *S. aureus* biofilms Tryptic soy broth was used. Incubation was done for 72 h in case of *E. coli* and *S. aureus* and 120 h in case of *P. aeruginosa* biofilms. The biofilms developed on coverslips were suspended in 0.9% NaCl after gently washing with PBS to remove planktonic cells. The suspended EPS (0.5 ml) was treated with the enzyme cocktail for 1 h at 37 °C for the degradation of the EPS. The EPS sample without any enzyme treatment and heat-inactivated enzyme cocktail served as control. Following incubation, the samples were taken for EPS analysis for the quantification of carbohydrate content (expressed as micrograms per milliliter). The amount of reducing sugar at different time intervals was determined using the DNS (dinitrosalicyclic) assay described by Miller (1959).

### Confocal laser scanning microscopy (CLSM)

For confocal microscopy, biofilms of the selected pathogens were developed on coverslips. After biofilm development, the coverslips were rinsed with PBS (0.05 M, pH 7.0) to take out the planktonic biomass. Thereafter, the coverslips were treated with the enzyme cocktail for 15 min at room temperature. Coverslips without enzymatic treatment served as control. The coverslips were then taken out, washed with PBS, and were stained for 15 min with SYTO 9® and propidium iodide using a LIVE/DEAD *Bac*light™ kit (Invitrogen). After staining, the excess stain was removed by washing with PBS. The coverslips were air-dried, placed on a glass slide, and visualized under a confocal microscope (Nikon).

### Sample preparation for field emission scanning electron microscopy (FESEM)

#### Biofilm on the tooth surface

Extracted teeth (waste material from a local hospital) were used for the preparation of dentin discs. Isomet low speed saw was used for sectioning the teeth and 2 mm thick cross-sections were prepared. The discs were exposed to 1% NaOCl for 15 min and then rinsed with distilled water. The discs were then autoclaved and kept in 12-well plate aseptically. 2 ml tryptic soy broth (TSB) (supplemented with 1% glucose) containing 10^5^ cells of *S. aureus* were added to the wells containing sterile dentin discs. Biofilm was allowed to form for 5 days at 37 °C. During the incubation period, the medium was changed daily. After the incubation period, PBS was used to gently wash the dentin discs to remove the planktonic cells. Following washing, the dentin discs were transferred to fresh wells and were treated with the enzyme cocktail for 15 min at room temperature. Dentin discs without enzymatic treatment served as controls. After the treatment, the test and control discs were processed for FESEM analysis as described by us earlier (Kaur et al. 2020a). Briefly, the test and the control discs were fixed with 2.5% glutaraldehyde for 2 h. Following fixation, glutaraldehyde was removed by washing thrice with 0.1 M PBS (pH 7.2). After fixation, gradual dehydration was performed by incubating the dentin discs with increasing concentration of ethanol (30–90%), for about 15 min in each gradient. Final dehydration was done with 100% ethanol at room temperature. The dehydrated samples were dried, gold-sputtered and examined under Field Emission Scanning Electron Microscope (SU8010, Hitachi).

#### Biofilm on Foley’s catheter

Biofilms of *E. coli* and *P. aeruginosa* were developed on 1 cm pieces cut from a sterile Foley catheter (Rusch). The biofilms were developed under static conditions for 7 days at 37 °C by the method of Mandakhalikar et al. (2018). Fed-batch culture method i.e. the growth medium (LB) was replaced with fresh medium after every 24 h. After establishing biofilm, the effect of enzyme treatment for its dispersal was assessed. After the establishment of the biofilm enzyme treatment for the biofilm dispersal was carried out for 15 min after which treated and control catheter samples were prepared for FESEM analysis as described above.

#### Biofilm on contact lens

*Staphylococcus aureus* biofilms were developed on contact lenses (Silkens Aquasoft) as well. Briefly, the contact lenses were kept in sterile 12-well plates and 5 ml TSB (supplemented with 1% glucose) containing 10^5^ *S. aureus* cells respectively were added to the wells containing sterile contact lenses. Biofilm was allowed to form for 2 days at 37 °C under static conditions. During the incubation period, the medium was changed daily. After the establishment of biofilm on the lenses, enzyme treatment for the biofilm dispersal was evaluated. Treated and control contact lenses were prepared for FESEM analysis as described above.

### Assessment of the enzyme cocktail in conjunction with antibiotics

#### Minimum inhibitory concentration (MIC)

MICs of the selected antibiotics were determined by the broth micro-dilution method, as per CLSI guidelines, and as described by us earlier (Singh et al. 2014). Briefly, the selected antibiotics i.e. ampicillin, ciprofloxacin, and chloramphenicol were added to the bacterial suspension containing approximately 10^7^ CFU/ml of log phase cells of the bacterial strains in a 96-well flat bottom plate. Plates were incubated at 37 °C for 18–24 h. Inhibition of bacterial growth was determined by comparing the change in turbidity at OD_600_ in the presence of the antibiotics to that in the absence of the agent.

#### Biofilm bactericidal concentration (BBC)

To determine the BBC values, biofilms were developed in a 96-well tissue culture microtiter plates. After the development of biofilms, planktonic cells were removed by washing three times with PBS and air-dried. Serial twofold dilutions of the antibiotics were prepared and added to corresponding wells (in the presence and absence of the enzyme cocktail). Plates were incubated for 24 h at 37 °C. After the incubation, antibiotics were aspirated gently, plates were washed two times with sterile PBS solution and wells were scraped thoroughly with a cell scraper, with particular attention to the well edges. 100 µl of different dilutions of the scrapped cell samples were plated on Mueller Hinton Agar (MHA) and incubated at 37 °C overnight. Colony-forming units (CFUs) were counted after 24 h. The BBC was defined as the lowest concentration of antibiotic that prevented bacterial re-growth (Fernández-Olmos et al. 2012).

#### Evaluation of effective concentration of antibiotics in the presence of enzyme cocktail

To study the effect of enzyme cocktail and antibiotics in combination, 100 μl of MHB was added into each well of the microtiter plates. Twofold serial dilutions of enzyme cocktail and antibiotics with concentrations ranging from 0.125 times MIC to 1 time the MIC were prepared. Different dilutions of the enzyme cocktail were added to the wells of a 96-well plate in a vertical orientation, and that of antibiotic was added in a horizontal orientation. Each well was supplemented with 10^6^ CFU/ml of the test strain. The final volume was made up to 200 μl in each well using PBS and the plate was incubated at 37 °C for 16–18 h. Wells without any antibiotic were used as positive growth controls and the susceptibility of the planktonic and sessile cells to the antibiotics was checked.

### Statistical analysis

For RSM, Design expert, version 7.0 was used for the experimental design and regression analysis of the experimental data. Statistical analysis of the model was performed to evaluate the analysis of variance (ANOVA). Quality of the polynomial model equation was judged statistically by the coefficient of determination R^2^ and its statistical significance was determined by F-test. The significance of regression coefficients was tested by a t-test. The data are expressed as mean ± standard deviation. Statistical analysis was done by Dunnett's multiple comparison tests. In all data analysis, p-values of 0.05 or less (p < 0.05) were considered significant.

## Results

Earlier we had reported enzyme optimization by classical one variable at a time (OVAT) approach (Kaur et al. 2020a). For the application of enzymes for biofilm dispersal, the yields of all the enzymes were further enhanced by evaluation of interactions between the most important variables promoting enzyme production by response surface methodology (RSM) studies.

### Statistical modelling by RSM for optimization of enzyme(s) production by *A. niger* APS

Out of the different paramateres screened, interaction between corn steep liquor and MgSO_4_ were observed to be most significant. Interactions between the selected parameters i.e. corn steep liquor (CSL), CaSO_4_, MgSO_4_ and tween 80 were analysed for all the enzymes in the cocktail. By applying multiple regression analysis on the experimental data, the following polynomial equations were derived for each enzyme:

$$\begin{aligned} {\text{CMCase}} & = + \,7.29 + 0.10 \times {\text{A}} - 0.016 \times {\text{B}} - 0.045 \times {\text{C}} - 0.089 \times {\text{D}} - 0.11 \times {\text{A}} \times {\text{B}} + 0.18 \times {\text{A}} \times {\text{C}} + 0.14 \times {\text{A}} \\ & \quad \times\, {\text{D}} + 0.049 \times {\text{B}} \times {\text{C}} + 0.12 \times {\text{B}} \times {\text{D}} - 0.13 \times {\text{C}} \times {\text{D}} - 0.016 \times {\text{A}}2 - 0.19 \times {\text{B}}2 - 0.21 \times {\text{C}}2 - \, 0.19 \times {\text{D}}2 \\ \end{aligned}$$

$$\begin{aligned} {\text{FPase }} & = +\, 2.14 + 0.031 \times {\text{A}} + 0.0069 \times {\text{B}} - 0.021 \times {\text{C}} - 0.028 \times {\text{D}} - 0.15 \times {\text{A}} \times {\text{B}} + 0.047 \times {\text{A}} \times {\text{C}} + 0.044 \times {\text{A}} \\ & \quad \times \,{\text{D}} + 0.15 \times {\text{B}} \times {\text{C}} + 0.035 \times {\text{B}} \times {\text{D}} - 0.035 \times {\text{C}} \times {\text{D}} - 0.053 \times {\text{A}}2 - 0.060 \times {\text{B}}2 - 0.063 \times {\text{C}}2 - \, 0.058 \times {\text{D}}2 \\ \end{aligned}$$

$$\begin{aligned}\upbeta {\text{-glucosidase}} & = +\, 6.29 + 0.10 \times {\text{A}} - 0.014 \times {\text{B}} - 0.063 \times {\text{C}} - 0.076 \times {\text{D}} - 0.056{\text{ A}} \times {\text{B}} + 0.015 \times {\text{A}} \times {\text{C}} + 0.12 \times {\text{A }} \\ & \quad \times \,{\text{D}} + 0.65 \times {\text{B}} \times {\text{C}} + 0.089 \times {\text{B}} \times {\text{D}} - 0.088 \times {\text{C}} \times {\text{D}} - 0.015 \times {\text{A}}2 - 0.017 \times {\text{B}}2 - 0.019 \times {\text{C}}2 - 0.017 \times {\text{D}}2 \\ \end{aligned}$$

$$\begin{aligned} {\text{Pectinase}} & = +\, 14.29 + 0.21 \times {\text{A}} - 0.040 \times {\text{B}} - 0.13 \times {\text{C}} - 0.16 \times {\text{D}} - 0.15{\text{ A}} \times {\text{B}} + 0.34 \times {\text{A}} \times {\text{C}} + 0.26 \times {\text{A}} \\ & \quad \times \,{\text{D}} + 0.14 \times {\text{B}} \times {\text{C}} + 0.22 \times {\text{B}} \times {\text{D}} - 0.23 \times {\text{C}} \times {\text{D}} - 0.36 \times {\text{A}}2 - 0.41 \times {\text{B}}2 - 0.44 \times {\text{C}}2 - 0.41 \times {\text{D}}2 \\ \end{aligned}$$

$$\begin{aligned} {\text{Mannanase}} & = + \,10.32 + 0.16 \times {\text{A}} - 0.054 \times {\text{B}} - 0.092 \times {\text{C}} - 0.12 \times {\text{D}} - 0.10{\text{A}} \times {\text{B}} + 0.25 \times {\text{A}} \times {\text{C}} + 0.20 \times {\text{A}} \\ & \quad \times\, {\text{D}} + 0.11 \times {\text{B}} \times {\text{C}} + 0.14 \times {\text{B}} \times {\text{D}} - 0.16 \times {\text{C}} \times {\text{D}} - 0.24 \times {\text{A}}2 - \, 0.32 \times {\text{B}}2 - 0.30 \times {\text{C}}2 - 0.28 \times {\text{D}}2 \\ \end{aligned}$$

$$\begin{aligned} {\text{Xylanase}} & = +\, 60.65 + 1.05 \times {\text{A}} - 0.13 \times {\text{B}} - 0.56 \times {\text{C}} - 0.68 \times {\text{D}} - 0.56{\text{ A}} \times {\text{B}} + 1.47 \times {\text{A}} \times {\text{C}} + 1.16 \times {\text{A}} \\ & \quad \times \,{\text{D}} + 0.67 \times {\text{B}} \times {\text{C}} + 0.85 \times {\text{B}} \times {\text{D}} - 0.90 \times {\text{C}} \times {\text{D}}-1.44 \times {\text{A}}2-1.64 \times {\text{B}}2-1.86 \times {\text{C}}2-1.67 \times {\text{D}}2 \\ \end{aligned}$$

$$\begin{aligned} {\text{Glucoamylase}} & = +\, 62.12 + 1.08 \times {\text{A}} - 0.017 \times {\text{B}} - 0.056 \times {\text{C}} - 0.74 \times {\text{D}} - 0.54{\text{A}} \times {\text{B}} + 1.44 \times {\text{A}} \times {\text{C}} + 1.18 \times {\text{A}} \\ & \quad \times\, {\text{D}} + 0.72 \times {\text{B}} \times {\text{C}} + 0.92 \times {\text{B}} \times {\text{D}} - 0.88 \times {\text{C}} \times {\text{D}}-1.43 \times A2-1.70 \times {\text{B}}2-1.94 \times {\text{C}}2-1.74 \times {\text{D}}2 \\ \end{aligned}$$

$$\begin{aligned} \upalpha {\text{-Amylase}} & = +\, 4518.67 + 73.85 \times {\text{A}}-11.24 \times {\text{B}}-43.90 \times {\text{C}} - 50.87 \times {\text{D}}-36.79{\text{A}} \times {\text{B}} + 111.53 \times {\text{A}} \times {\text{C}} + 86.75 \times {\text{A }} \\ & \quad \times\, {\text{D}} + 41.78 \times {\text{B}} \times {\text{C}} + 65.21 \times B \times {\text{D}}- 64.11 \times {\text{C}} \times {\text{D}}-109.47 \times {\text{A}}2-124.16 \times {\text{B}}2-136.78 \times {\text{C}}2-124.26 \times {\text{D}}2 \\ \end{aligned}$$

$$\begin{aligned} {\text{Alginate}}\;{\text{lyase}} & = +\, 5.62 + 0.11 \times {\text{A}} - 0.013 \times {\text{B}} - 0.035 \times {\text{C}} - 0.063 \times {\text{D}} - 0.059{\text{A}} \times {\text{B}} + 0.12 \times {\text{A}} \times {\text{C}} + 0.11 \times {\text{A}} \\ & \quad \times \,{\text{D}} + 0.082 \times {\text{B}} \times {\text{C}} + 0.087 \times {\text{B}} \times {\text{D}} - 0.071 \times {\text{C}} \times {\text{D}}-0.12 \times {\text{A}}2 -0.15 \times {\text{B}}2-0.17 \times {\text{C}}2 -0.15 \times {\text{D}}2 \\ \end{aligned}$$

F values of 719.03, 167.83, 256.41, 195.36, 261.37, 541.85, 294.82, 50,586.66 and 295.66 for CMCase, FPase, β-glucosidase, pectinase, mannanase, xylanase, glucoamylase, α-amylase and alginate lyase respectively imply that the model was significant. In case of all the enzymes, the values of “Prob > F” were less than 0.05 which indicated that the model terms were significant. The "Pred R-Squared" were in reasonable agreement with the "Adj R-Squared" and the value of "Adeq Precision" which measures the signal to noise ratio > 4 was found to be desirable. These results indicated that the model was statistically sound and suitable to navigate the design space. To determine the optimal levels of the variables, 2D contour plots for all the enzymes were constructed. However, representative plots of the most significant variables i.e. CSL and MgSO_4_ are shown in Additional file 1: Fig. S1.

To evaluate accuracy of the statistical experimental model of RSM, attempts were made to formulate a common medium for maximizing the yields of all the enzymes using different variables selected for the studies. Numerical optimization for enzymes(s) production attempted with Design-Expert version 7.0 using A (corn steep liquor 0.086%), B (CaSO_4,_ 0.019%), C (MgSO_4_, 0.020) and D (tween-80, 0.06%), in wheat bran (1.7%) and kitchen waste (1.88%) based medium supplemented with CMC (0.56%), de-oiled rice bran (0.70%), peptone (0.76%), KH_2_PO_4_ (0.02%), FeSO_4_, (0.02%), inoculated with 4 fungal discs and incubated at 30 °C for 6 days under static conditions predicted the yields of 7.314 IU/ml, 2.13 IU/ml, 6.31 IU/ml, 10.36 IU/ml, 60.87 IU/ml, 14.33 IU/ml, 62.36 IU/ml, 4534 IU/ml, 5.64 IU/ml for CMCase, FPase, β-glucosidase, mannanase, xylanase, pectinase, glucoamylase, α-amylase and alginate lyase respectively. To validate the optimum concentrations, an experiment with the above-specified conditions was performed and the results were 7.17 IU/ml, 2.07 IU/ml, 6.08 IU/ml, 10.16 IU/ml, 59.38 IU/ml, 13.27 IU/ml, 60.24 IU/ml, 4472 IU/ml and 5.37 IU/ml for CMCase, FPase, β-glucosidase, mannanase, xylanase, pectinase, glucoamylase, α-amylase, and alginate lyase respectively. Observed results were found to be in agreement with the predicted result values hence validating the model.

### Characterization of individual enzyme present in the cocktail obtained from *A. niger* APS

#### Temperature activity profile

**Fig. 1:**
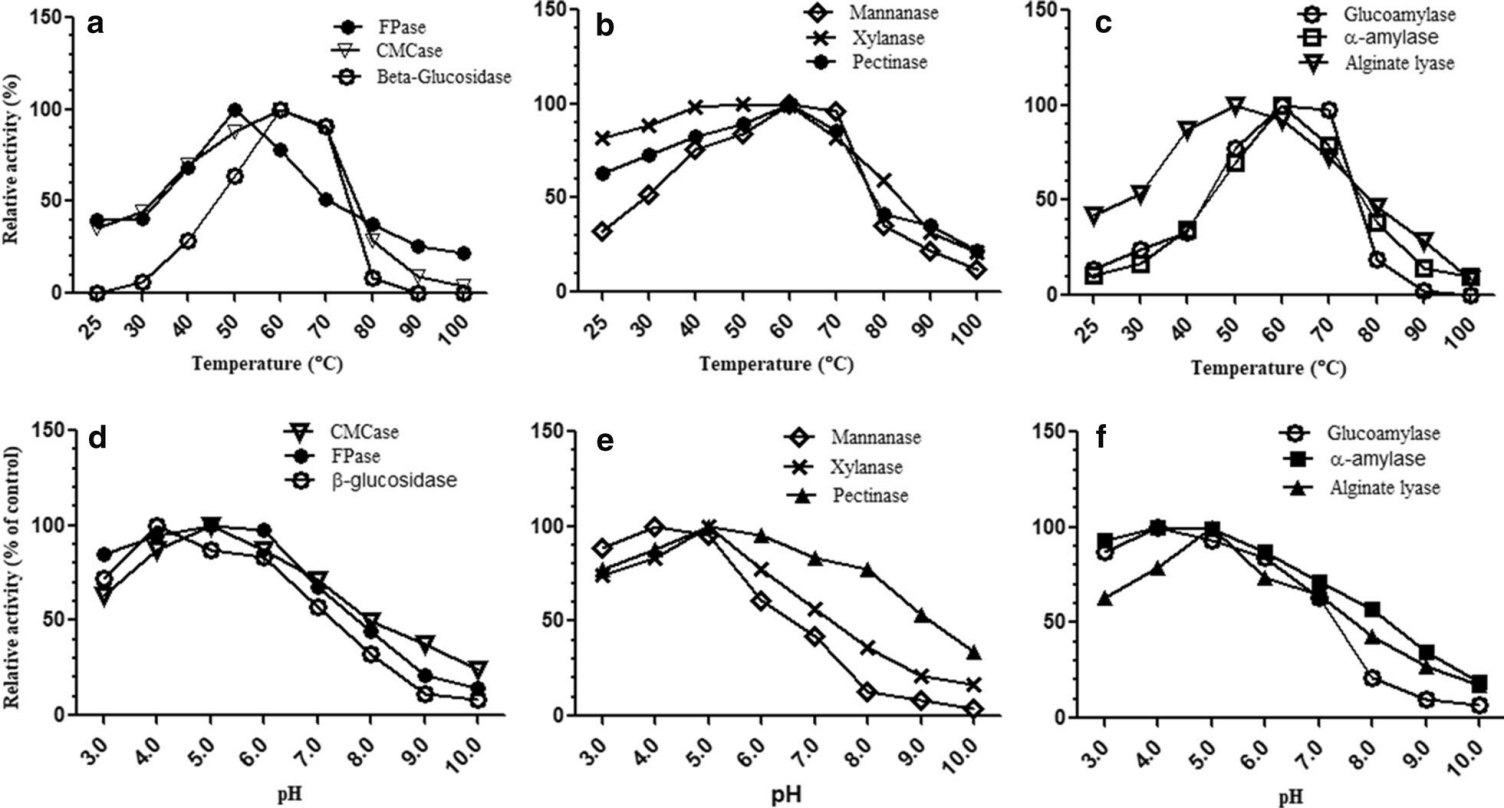
Temperature versus activity profiles (**a**–**c**) and pH versus activity profiles (**d**–**f**) of different enzymes obtained from *A. niger* APS. Control is the maximum activity exhibited by an enzyme at a particular temperature or pH. All the enzymes in the cocktail were found to be active over a wide range of temperature and pH

The results showed that all the enzymes in the cocktail had compatible temperature optimas and the enzymes were active over a broad temperature range. The temperature optima for CMCase, β-glucosidase, mannanase, glucoamylase, α-amylase and pectinase was found to be 60 °C while in case of FPase, xylanase and alginate lyase the temperature optima was 50 °C. The results are shown in Fig. 1.

#### pH activity profile

All the enzymes in the cocktail were found to be active over a wide pH range. In case of CMCase, FPase, xylanase, pectinase and alginate lyase maximum activity was revealed at pH 5.0. The enzymes were appreciably active in the pH range of 4.0–6.0. The temperature optima’s for β-glucosidase, mannanase, α-amylase and glucoamylase was observed at pH 4.0. However, with rise in pH, the activities decreased and a steep fall in enzyme activities was observed beyond pH 6.0. The results are depicted in Fig. 1.

#### Thermostability profiles

**Fig. 2:**
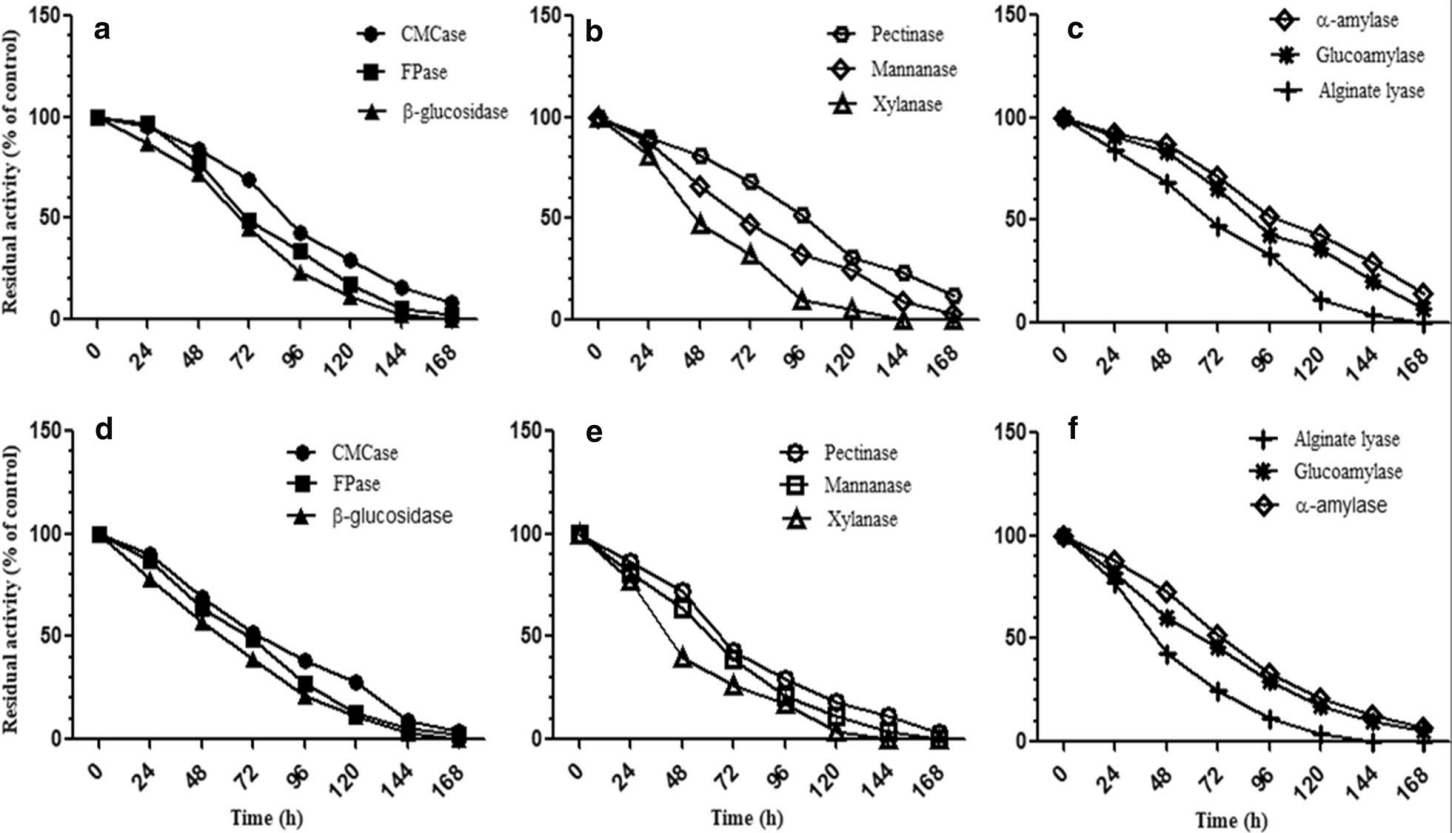
Thermostability profiles of different enzymes from surface cultures of *A. niger* APS at 50 °C (**a**–**c**) and at 60 °C (**d**–**f**) in 0.1 M acetate buffer, pH 4.0. The residual activity was determined by the standard assay under normal conditions and expressed in terms of % of control. The enzymes in the cocktail were found to have appreciable thermostability

The enzymes were found to be appreciably thermostable at 50 °C and retained residual activity even after 168 h. The T_1/2_ lives of CMCase, FPase and β-glucosidase at 50 °C were 52 h, 70 h and 68 h respectively. Pectinase was found to be more thermostable with T_1/2_ of 98 h and retained 12% residual activity even after 168 h of incubation at 50 °C. T_1/2_ life of mannanase was found to be 69 h at 50 °C. As compared to other enzymes, xylanase was less thermostable with T_1/2_ life of only 47 h. T_1/2_ of α-amylase and glucoamylase was 97 h and 94 h respectively showing the thermostable nature of these enzymes. Half life of alginate lyase was found to be 70 h. The enzymes were found to be less thermostable at 60 °C as compared to 50 °C and half lives of CMCase, FPase, β-glucosidase, pectinase, mannanase, xylanase, α-amylase, glucoamylase and alginate lyase were 82 h, 70 h, 68 h, 67 h, 66 h, 38 h, 70 h, 67 h ad 46 h respectively. The results are shown in Fig. 2.

#### pH stability profiles

**Fig. 3:**
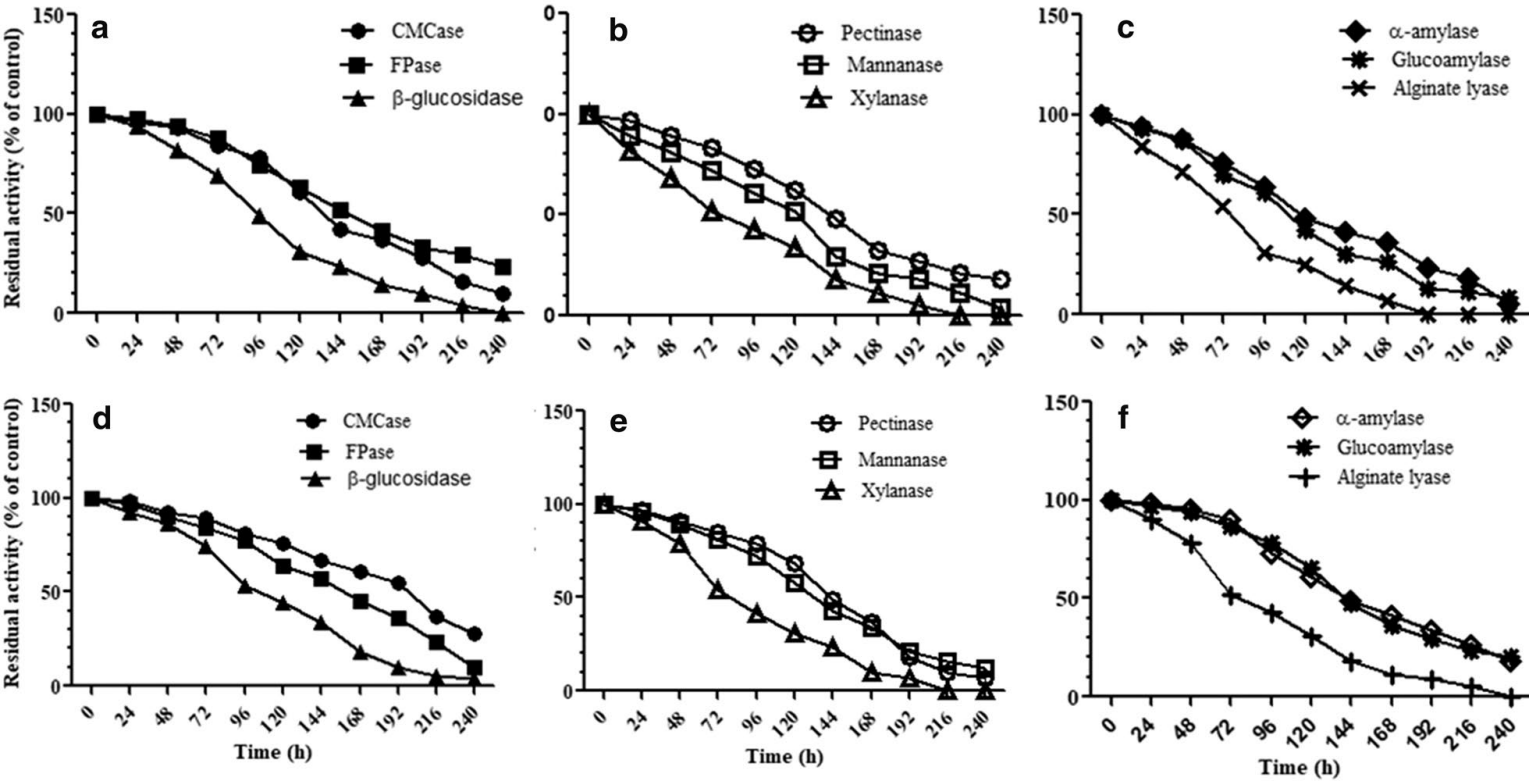
pH-stability profiles of different enzymes from surface cultures of *A. niger* APS at pH 4.0 (**a**–**c**) and at pH 5.0 (**d**–**f**). The samples were withdrawn at 0, 2, 4, 6, 24 h and the residual activities were determined at 50 °C and expressed in terms of % of control. Enzymes in the cocktail were found to be highly pH stable under acidic conditions (pH 4–5)

The enzymes were found to be highly pH stable under acidic conditions. At pH 4.0, the half lives of CMCase, FPase, β-glucosidase, pectinase, mannanase, xylanase, α-amylase, glucoamylase and alginate lyase were 132 h, 148 h, 95 h, 145 h, 123 h, 74 h, 121 h, 111 h and 74 h respectively. At pH 5.0, the half lives of CMCase, FPase, β-glucosidase, pectinase, mannanase, xylanase, α-amylase, glucoamylase and alginate lyase were 198 h, 165 h, 102 h, 144 h, 131 h, 76 h, 146 h, 148 h and 76 h respectively. The results are shown in Fig. 3.

#### Effect of metal salts, surfactants and EDTA on enzyme activities

**Fig. 4:**
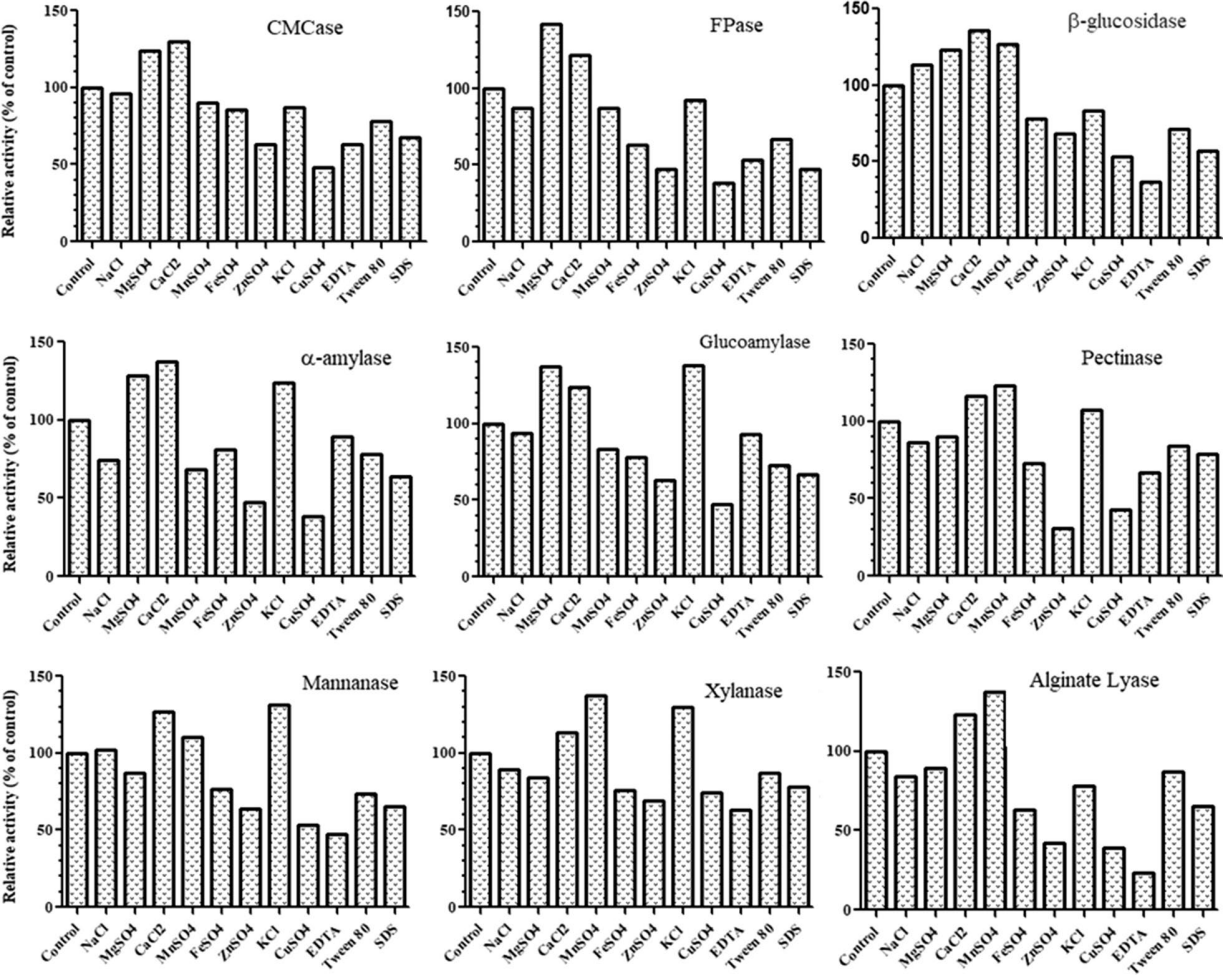
Effect of various metal salts, EDTA and surfactants on activities of different enzymes obtained from surface cultures of *A. niger* APS

The results (Fig. 4) revealed that MgSO_4_ and CaCl_2_ enhanced the activity of CMCase and FPase while FeSO_4_, ZnSO_4_ and CuSO_4_ had significant inhibitory effect on the enzyme activity. In case of β-glucosidase, MgSO_4_, CaCl_2_, NaCl and MnSO_4_ had stimulatory effect while other metal ions led to a decrease in the activity. MnSO_4_, KCl and CaCl_2_ enhanced the xylanase and mannanase activity. Use of MgSO_4_, CaCl_2_ and KCl had a stimulatory effect on activities of amylases. CaCl_2_, KCl and MnSO_4_ increased the pectinase activities while other metal ions had inhibitory or no significant effect. CaCl_2_, MnSO_4_ had a positive effect in alginate lyase activity as compared to the control. EDTA was found to significantly reduce the activities of cellulases hemicellulases, pectinase and alginate lyase while no significant effect in glucoamylase and α-amylase was observed. Similarly, the surfactants were also found to have inhibitory effect on the enzyme activities.

### Composition assessment of the enzyme cocktail

The enzyme cocktail obtained from surface cultures of *A. niger* APS, was checked for the presence of other enzymes (protease and lipase) in the supernatant. As compared to the control wells containing commercial proteases, the zone of hydrolysis was very small around wells containing the enzyme cocktail and neutralized enzyme cocktail. Further, on comparing the neutralized enzyme cocktail and enzyme cocktail, the zone around the former was much smaller than the later, indicating that low pH of the enzyme cocktail (pH 4.5) also resulted in the zone formation around the wells (Additional file 1: Fig. S2a). Further, the protease activity detected using casein as substrate confirmed the presence of only 0.166 IU/ml protease activity in the enzyme cocktail obtained from *A. niger* APS. Lipase activity was found to be absent in the enzyme cocktail as no zone of hydrolysis was observed around the wells containing the enzyme cocktail which was also confirmed using an auto-analyzer. In contrast, clear zones of hydrolysis were observed around the positive control wells, containing different dilutions of the lipase enzyme (Additional file 1: Fig. S2b).

### Assessment of the antibiofilm potential of the in-house produced enzyme cocktail

**Fig. 5:**
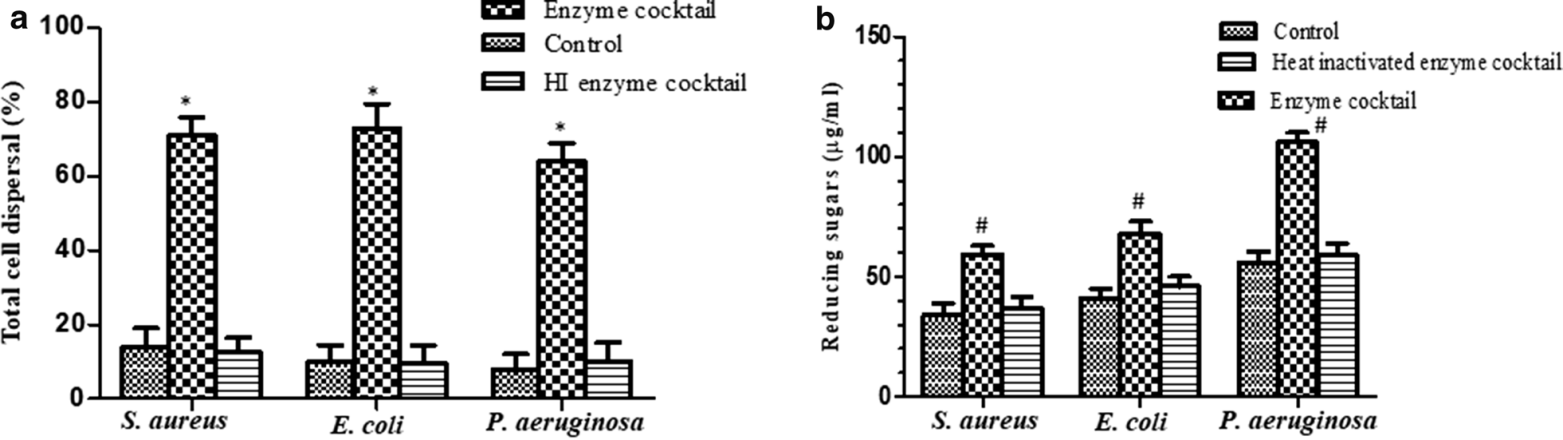
**a** Total cell dispersal from *S. aureus*, *E. coli*, and *P. aeruginosa* biofilms after treatment with multiple carbohydrases *p < 0.001 enzyme treatment vs control and heat inactivated enzyme cocktail; **b** amount of reducing sugar released from biofilm EPS after enzyme treatment ^#^p < 0.01 enzyme treatment vs. control and heat inactivated enzyme cocktail

**Fig. 6:**
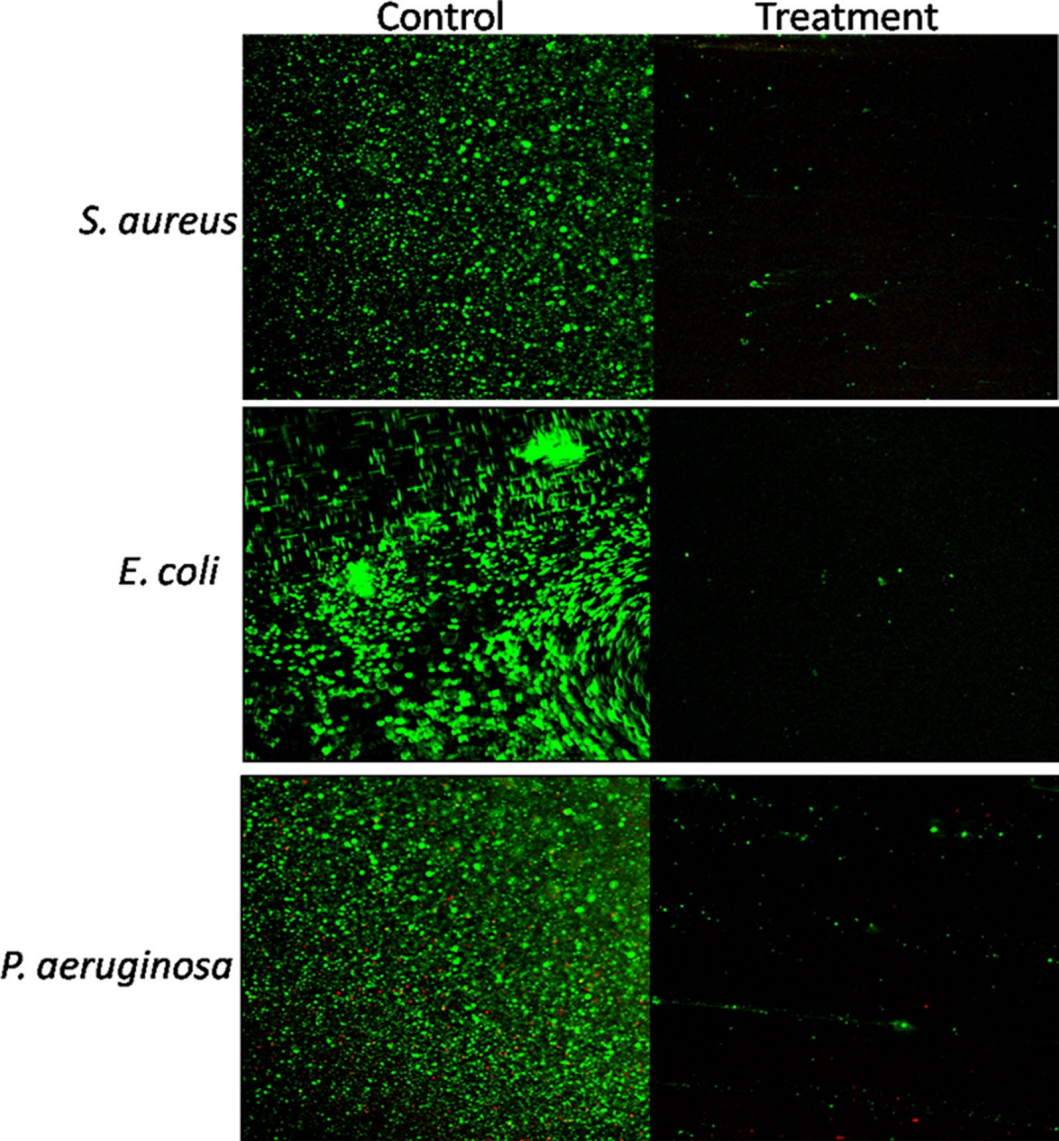
CLSM images of biofilms before and after treatment with enzyme cocktail. Green coloured cells represent live cells whereas red coloured cells are dead cells. A mat of live cells was observed in control whereas after treatment with enzyme cocktail, very few live cells were visible due to biofilm disruption

An in-vitro cell dispersal assay was performed to estimate the total dispersal of cells from the biofilms of selected pathogens by the action of enzymes. Percentage of total cells dispersed from the biofilms after the enzyme treatment was calculated. Significant dispersal (p ≤ 0.001) of the cells from biofilms was observed in case of all the pathogens i.e. *S. aureus*, *E. coli*, and *P*. *aeruginosa* as compared to the controls (PBS and heat-inactivated enzymes) (Fig. 5a). Since, a significant number of CFUs after the enzymatic treatment could be recovered in the supernatant, it can thus be inferred that the enzymes do not have bactericidal activity but simply disperse the biofilm cells into their planktonic state by disrupting the biofilm architecture. Further, the ability of enzyme cocktail to act upon the polysaccharides present in the biofilm EPS was also determined. As shown in Fig. 5b, a significant increase (p ≤ 0.01) in the reducing sugar content was observed after enzyme treatment as compared to the control indicating the degradation of EPS present in the biofilm. The effect of multiple carbohydrases on biofilm architecture and cell viability was further substantiated by CLSM and FESEM studies. In Fig. 6, CLSM images revealed intact biofilms of all the pathogens viz, *S. aureus*, *E. coli*, and *P. aeruginosa* in the control. In between the mat of live green cells, few red cells were also visible which could be the dead cells or the extracellular DNA (eDNA) present in the biofilm EPS. However, in case of biofilms treated with enzymes, the biofilm structure was completely lost and few live cells (seen as a small cluster of green cells) were observed.

**Fig. 7:**
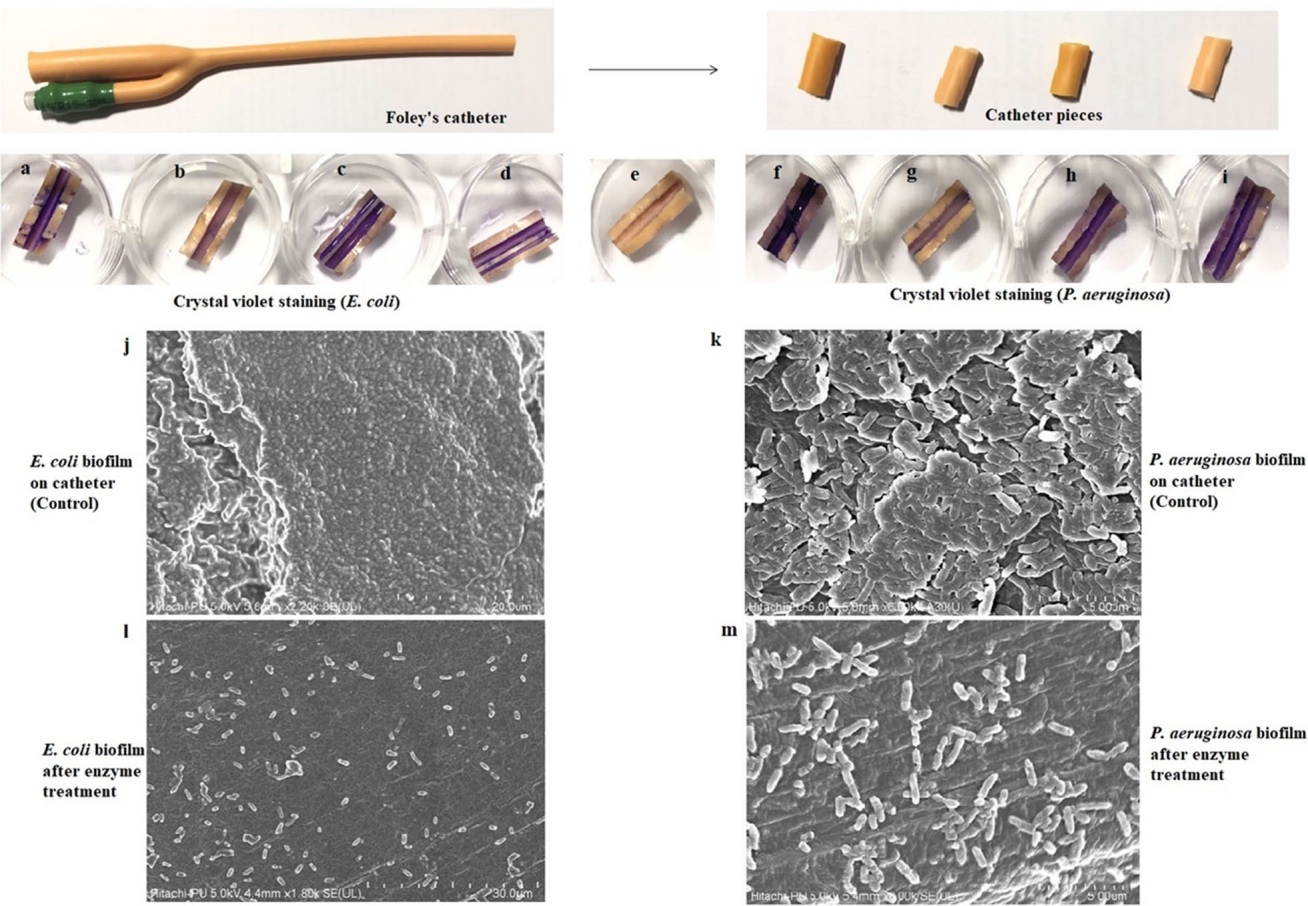
Effect of in-house produced enzyme cocktail on *E. coli* and *P. aeruginosa* biofilms developed on Foley’s catheter. The panel shows the placement of crystal violet stained pieces cut from Foley’s catheter wherein (**a**–**d** biofilm produced by *E. coli*). **a** Control (untreated); **b** treated with enzyme cocktail; c treated with heat-inactivated enzymes; **d** treated with PBS; **e** control (no biofilm, no enzyme treatment); (**f**–**i** biofilm produced by *P. aeruginosa*). **f** Control (untreated); **g** treated with enzyme cocktail; **h** treated with heat-inactivated enzymes; **i** treated with PBS. **j** FE-SEM images of the (untreated) biofilms of *E. coli* and **k** *P. aeruginosa* established on Foley’s catheter, **l** disrupted *E. coli* and **m** *P. aeruginosa* biofilms after treatment with in-house produced enzyme cocktail. No difference was observed between heat inactivated, PBS treated and positive controls

**Fig. 8:**
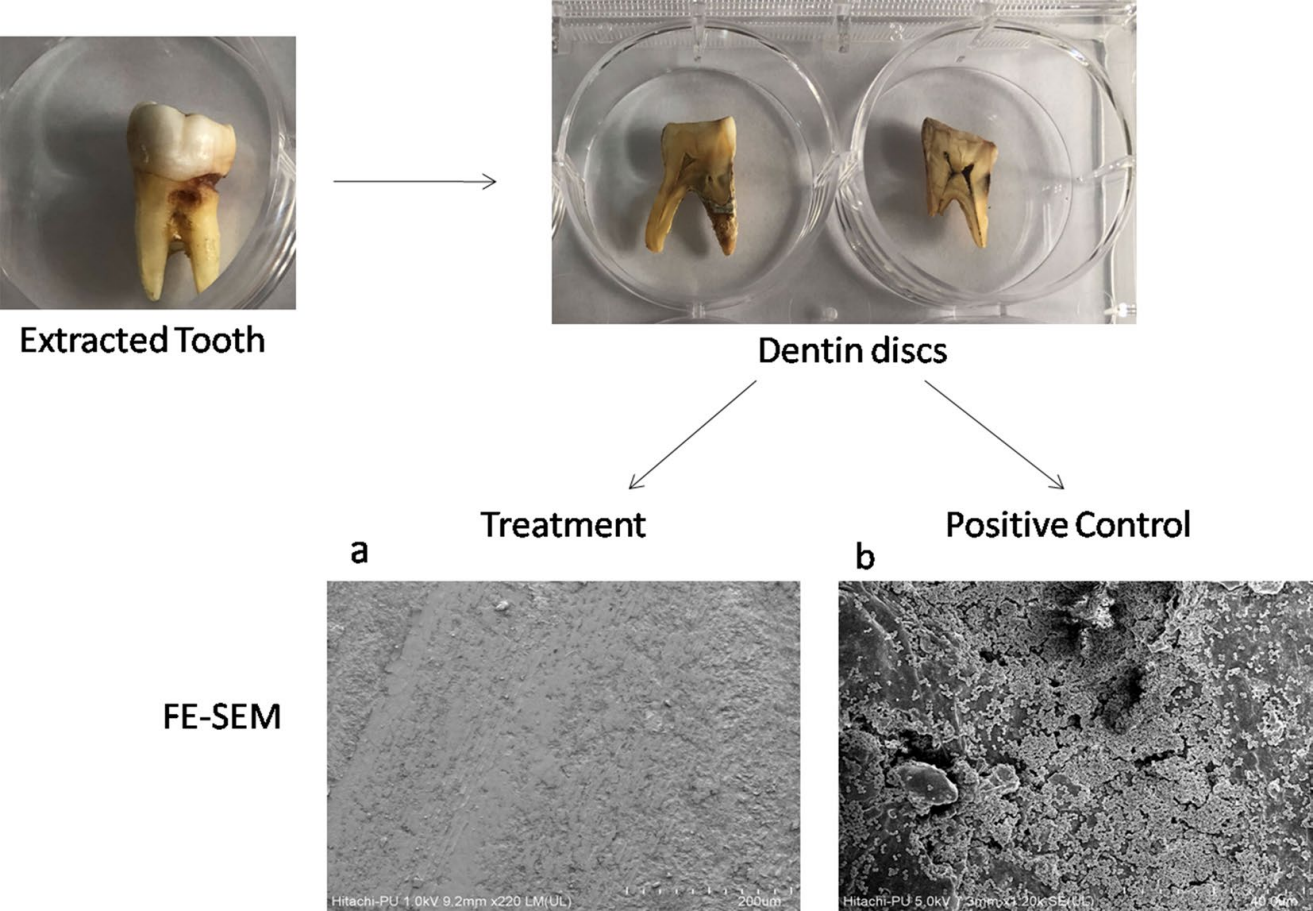
FE-SEM images of the *S. aureus* biofilms developed on dentin disc, **a** after treatment with in-house produced enzyme cocktail and **b** control (untreated) biofilms. Confluent growth of biofilms with typical biofilm architecture was seen in all the controls while biofilm dispersal was observed after enzymatic treatment

**Fig. 9:**
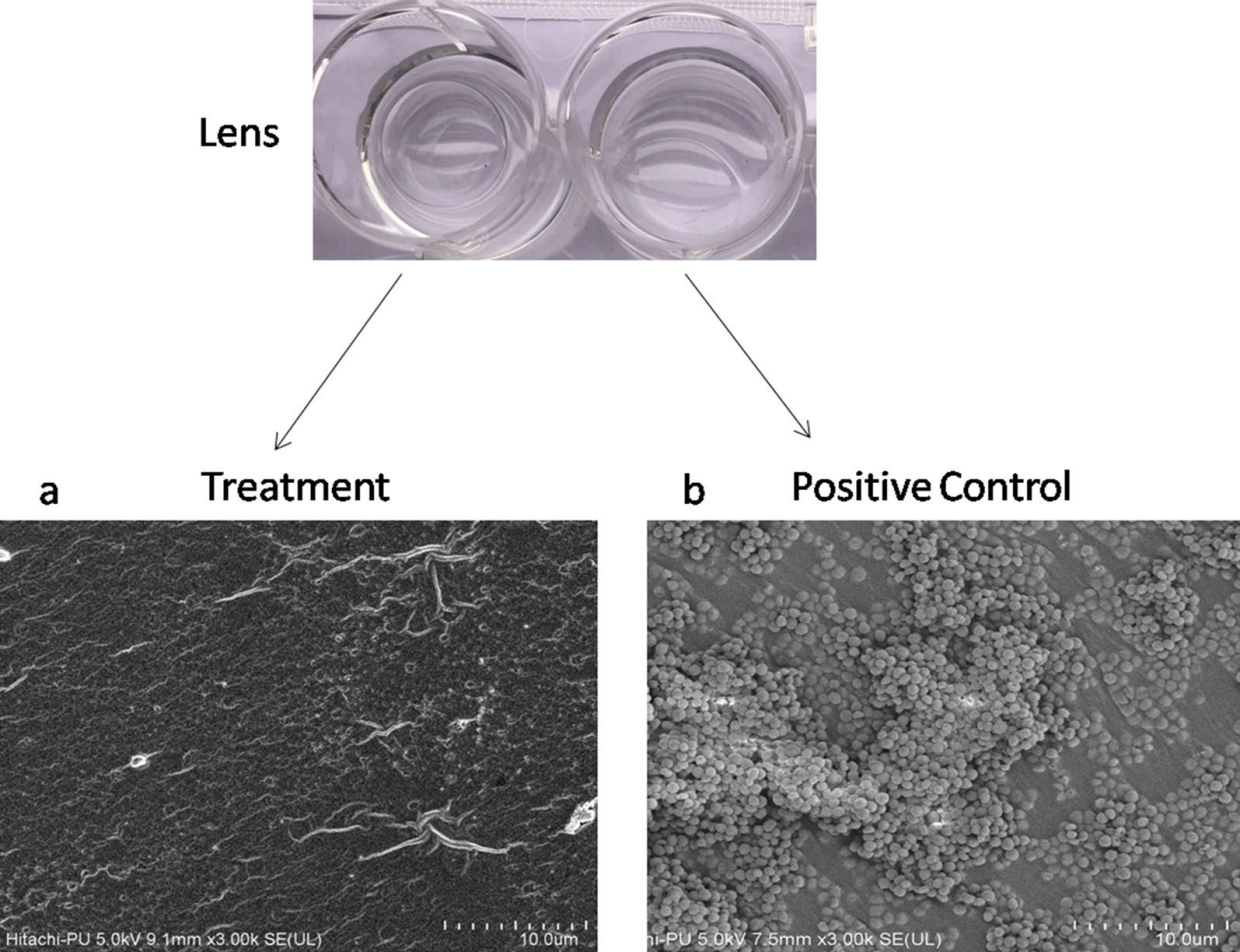
FE-SEM images of the *S. aureus* biofilms developed on contact lens, **a** after treatment with in-house produced enzyme cocktail and **b** control (untreated) biofilms. Confluent growth of biofilms with typical biofilm architecture was seen in all the controls while biofilm dispersal was observed after enzymatic treatment

Further, validation of the effect was done by FESEM, using various surfaces on which biofilm was earlier developed. As observed in Figs. 7, 8, and 9, large parts of the biofilms were found to be dispersed by the action of the multiple enzymes, while confluent growth of biofilms with typical biofilm architecture was observed in all the controls. Cells embedded within the biofilm EPS were seen in control while in enzyme-treated samples, single cells or very small bacterial aggregates were observed. In case of dentin discs and contact lenses, the surfaces were found to be totally clear after the enzyme treatment. FESEM analysis also confirmed that the carbohydrases act as dispersal agent and detach the cells encased within the biofilms.

### Evaluation of effective concentration of antibiotics in the presence of enzyme cocktail

In the present study, the potential of using enzymes in conjunction with antibiotics was assessed. MICs and BBCs of three antibiotics viz. ampicillin, ciprofloxacin, and chloramphenicol were checked against the selected pathogens in the presence and absence of the enzyme cocktail (Table 2). The MIC values of all the three antibiotics were significantly lowered in the presence of the enzyme cocktail. As anticipated, the BBC values of the tested antibiotics were found to be much higher than their MIC values against the same pathogen. However, it can be inferred from results that the use of the enzyme cocktail augmented the efficacy of the antibiotics by lowering both the BBC and corresponding MICs.

**Table 2:** Antibiotic susceptibilities of the planktonic cells and biofilm cells of *E. coli*, *S. aureus,* and *P. aeruginosa* in the absence and presence of the enzyme cocktail

Antibiotics (µg/ml)	Without enzyme cocktail (MIC)	With enzyme cocktail	Without enzyme cocktail (BBC)	With enzyme cocktail
	Planktonic bacteria		Biofilm bacteria	
	*E. coli*		*E. coli*	
Ampicillin	8.0	2.0	128	32
Ciprofloxacin	4.0	2.0	64	16
Chloramphenicol	8.0	4.0	64	32
	*S. aureus*		*S. aureus*	
Ampicillin	1.0	0.25	64.0	8.0
Ciprofloxacin	4.0	0.50	32.0	8.0
Chloramphenicol	8.0	2.0	32.0	16.0
	*P. aeruginosa*		*P. aeruginosa*	
Ciprofloxacin	16	8	128	64
Chloramphenicol	32	4	64	32

## Discussion

Taking into the consideration the heterogeneous nature of polysaccharides found in the biofilm EPS and intra-species and inter-species interactions of pathogens under the natural conditions, in the present study a cocktail of carbohydrases was used to disperse the biofilms of selected pathogens wherein the enzymes were expected to degrade the polysaccharides present in the biofilm EPS. For the application of enzymes for biofilm dispersal, the yields of all the enzymes produced by *A. niger* APS were optimized using RSM.

In our earlier study (Kaur et al. 2020a, b), we have reported the effect of various cultural and environmental factors by ‘one variable at a time’ (OVAT) method. However, when these factors are used in combination, then one factor might influence the effect of other factor. Therefore, statistical tools like RSM are very useful and widely employed for identifying the major independent variables or factors and their interactions among other factors that have a significant effect on a particular response (Khoshnam et al. 2019; Bazargan et al. 2020). Based on our earlier experiments, in the present study, interactions among four selected parameters i.e. corn steep liquor (CSL), CaSO_4_, MgSO_4_ and tween 80 were analysed for all the enzymes in the cocktail. This statistical tool was used to evaluate the nature of the response surface in the experimental region and to investigate the first and higher order main effects of each variable and interactions amongst them for further optimization of enzyme(s) production by *A. niger* APS under surface fermentation in media comprising wheat bran and kitchen waste. The variables were studied at five different levels as very low (− 2), low (− 1), centre (0), high (+ 1) and very high (+ 2). The respective effect of selected variables is explained by the values of coefficient estimates and final model equation for different enzymes. The graphical representation of the regression equations for all the enzymes is presented in the form of contour plots, indicating the interactions between two factors at a time. In the contour graphs, it can be observed that yield of particular enzyme is enhanced at certain concentrations of the selected variables, above and below these concentrations the enzyme yields tend to decrease. Finally, the accuracy of statistical model of RSM design, predicted by the software, was validated by conducting experiments with the specified conditions for all the enzymes individually as well as for all the enzymes in the cocktail. The observed increment in the enzyme productivities with the use of RSM might be due to the fine-tuning of medium components resulting in better growth of *A. niger* APS thereby enhancing the yields of all the enzymes in the cocktail. Overall optimization led to an increment of 3.32, 7.29, 3.64, 3.71, 2.56, 1.4, 3.82, 18.86 and 2.65-folds in the productivities of CMCase, FPase, β-glucosidase, pectinase, mannanase, xylanase, glucoamylase, α-amylase, and alginate lyase respectively as compared to the initial un-optimized medium. Earlier also these statistical tools have been employed for the fine-tuning of media components for the augmentation of enzyme productivities. Response surface methodology has been successfully applied for optimization of media components and culture conditions for the production of enzymes (Soni et al. 2012; Rana et al. 2013; Cotârleţ 2013; Rose et al. 2019), amino acids (Xiong et al. 2005), ethanol production (Karuppaiya et al. 2009).

Optimum temperature and pH are critical factors for the application of enzymes. Therefore, the enzymes in the cocktail were characterized in terms of temperature and pH activity profiles. All the enzymes in the cocktail had compatible temperature optimas and the enzymes were active over a broad temperature range. Earlier also cellulases and hemicellulases from various fungi have been reported to have a temperature optima between 50 and 65 °C e.g. mannanase from *Trichoderma* *reesei* and *Rhizopus* *oryzae* (Kupski et al. 2014), *Lichtheimia ramose* (Garcia et al. 2018), *Aspergillus niger* P-19 (Kaur et al. 2020b). Majority of cellulases and xylanases from thermophiles have been reported to have temperature optima between 50 and 80 °C (Bala and Singh 2019). The enzymes were also found to be appreciably thermostable 50 and 60 °C. Endoglucanase, β-glucosidase and xylanase from *A. niger* strain were found to have half lives of 43 h, 148 h and 90 h respectively (Farinas et al. 2010). Recently, a study carried in our laboratory reported highly stable cellulases and hemicellulases from *A. niger* P-19, retaining residual activity up to 408 h at 50–60 °C (Kaur et al. 2020b). Like temperature, pH also greatly affect the enzyme activities, since any change in pH results in alteration in the ionic character of enzyme which in turn affects both the catalytic site and conformational status of the enzyme (Prakash et al. 2009). Sinitsyn et al. (2014) reported optimal cellulose and hemicellulase activities from *Penicillium verruculosum* in the pH range of 3.5 to 5.5. Cellulases and hemicellulases produced by *Lichtheimia ramosa* and *A. niger* P-19 were found to be optimally active in the pH range of 4.5–5.5 (Garcia et al. 2018) and pH 4.0 (Kaur et al. 2020b) respectively. In our study, all the enzymes in the cocktail were found to be active over a wide pH range, which is a favourable finding as the cocktail can be utilized for biofilm dispersal under diverse conditions. Other factors that affect enzyme activities include metal ions, surfactants and chelators. Metal ions have been reported to enhance as well as reduce the activities of enzymes. Cu^2+^ and Fe^2+^ were reported to enhance endoglucanase activity obtained from *A. niger* VTCC-F021 while K^+^, Ca^2+^, Co^2+^, Mn^2+^, Zn^2+^, Ni^2+^, and Ag^+^ were found to decrease the endoglucanase activity (Pham et al. 2012). In another study carried by Vasconcellos et al. (2016), Co^2+^, Mn^2+^, Fe^2+^ (2 mM) enhanced endoglucanase activity. In the same study, Co^2+^ was found to enhance β-glucosidase from *A. niger* while Fe^2+^ strongly inhibited the activity. Cu^2+^, Co^2+^, Fe^2+^, Fe^3+^, Pb^2+^, Mg^2+^ have been reported to be inhibitory for xylanase activity (Ghanem et al. 2000; Vasconcellos et al. 2016). Similar to our results, EDTA was found to be inhibitory for endoglucanase and xylanase activities of *A. niger* (Vasconcellos et al. 2016).

The presence of other enzymes (protease and lipase) were also checked in the enzyme cocktail to rule out their role in the biofilm removal. The results suggested the presence of very low levels of protease in the cocktail while lipase activity was found to be completely absent. It could be inferred that proteases were produced/excreted extracellularly in very low amounts while lipase was not produced/excreted extracellularly by *A. niger* APS, suggesting that the observed biofilm dispersal was due to the presence of carbohydrases present in the enzyme cocktail in appreciable levels.

A significant dispersal of the biofilms was observed after the enzyme action. The cell dispersal assay revealed that a significant number of CFUs after the enzymatic treatment could be recovered in the supernatant. From these results it could be inferred that the enzymes do not have bactericidal activity but simply disperse the biofilm cells into their planktonic state by disrupting the biofilm architecture. These results are in concordance with our earlier study (Kaur et al. 2020a, b) wherein a significant reduction in the biofilm-biomass (based on crystal violet assay) was recorded after enzyme treatment. Other workers have also reported dispersal of biofilms by enzymatic action and EPS degradation (Kalpana et al. 2012; Fleming and Rumbaugh 2018). The action of multiple enzymes on biofilms was further substantiated by CLSM studies. The observation further confirms the mode of action of these enzymes which points towards degradation by cleaving particular glycoside linkages of the polysaccharides present in the biofilm EPS, resulting in biofilm dispersal to their planktonic state. The results are in consonance with earlier studies wherein these hydrolytic enzymes have led to the dispersal of biofilms by acting on the polysaccharides present in the biofilm EPS (Loiselle and Anderson 2003; Kalpana et al. 2012; Fleming et al. 2017).

Continual use of medical devices is often associated with increased risk of infections (Marrie et al. 1982; von Eiff et al. 2005; Stewart and Bjarnsholt 2020). The term ‘polymer-associated infection’ (Percival et al. 2015), has been coined for medical device-related infections which are associated with high morbidity and mortality. In this context, newer approaches are continuously being explored due to the inefficacy of current antimicrobial approaches to efficiently treat biofilm-associated infections. *S. aureus* is not only a major cause of various localized and systemic infections but also has been reported to have a strong connection to tooth decay and dental implant infections (Harris and Richards 2004; Salvi et al. 2008). Incidence of microbial biofilm formation by *S. aureus* on contact lenses and lens storage cases are also often encountered resulting in increased risk factors for contact lens-associated corneal infections. Another concern is the development of biofilms on the lumen and outer surface of the catheters. The microorganisms that colonize the periurethral skin are contaminants as these microorganisms migrate into the urinary bladder and are often associated with urinary tract infections (UTI) (Stickler 2008; Taheri et al. 2016). Almost 80% of nosocomial urinary tract infections (UTIs) are due to catheterization. Among various pathogens, *E. coli* and *P. aeruginosa* are most frequently associated with catheter associated-UTI (CAUTI) (Maharjan et al. 2018).

To combat medical devices related infections, one of the approaches is to disperse the components of the EPS matrix which acts as a ‘cementing material for the biofilms’. In the present study, prospect of using multiple enzymes for dispersal of biofilms from different surfaces including dental disc, contact lens, and Foley’s catheter was evaluated. Significant clearance of the biofilms developed on the above mentioned polymeric surfaces may be attributed to the loss of physical integrity of the biofilms after the degradation/hydrolysis of the polymers present in the biofilm EPS by the action of multiple carbohydrases present in the enzyme cocktail.

Since the use of enzymes is a non-bactericidal approach (Fleming et al. 2017), there is a risk that the cells dispersed from the mature biofilm by enzyme action might lodge into a new surface, ensuing infection therein. The large-scale dispersal of biofilms in a living host can overburden the immune system and might result in dissemination of the infection culminating in septicemia. In a study performed by Fleming and Rumbaugh (2018), large-scale, in-vivo dispersal of biofilm bacteria by enzymes (glycoside hydrolases) resulted in lethal septicemia in murine wound model in the absence of antibiotic treatment. It is also evident from the results of our study that the enzymes are not bactericidal in action but only result in dispersal of biofilm cells into their planktonic state probably due to loss of biofilm architecture. Therefore, in the present study we also assessed the potential of using the enzymes in conjunction with antibiotics. The biofilm bactericidal concentration (BBC) of the antibiotics was found to be much higher than the corresponding MIC values, which might be due to the protection provided by the EPS to the cells encased within the biofilm. It can be inferred from results that the use of enzyme cocktail augmented the efficacy of the antibiotics by lowering both the BBC and corresponding MICs. The reduced effective concentration of the different antibiotics in the presence of the enzyme cocktail might be attributed to the breakdown of the EPS which act as a physical barrier for the diffusion of the antibiotics into the biofilm. The reduced effective concentration of antibiotics may not only help in minimizing the chances of emergence of resistance but may also reduce the level of toxic effects associated with the antibiotics.

This study provides a bipartite approach; firstly, for the sustainable production of a consortium of carbohydrases from *A. niger*, secondly, it also gives an insight on the prospects of using multiple carbohydrases for the management of biofilms formed on the surfaces of medical devices. The study also suggests the possibility of using enzymes as an adjunct to antibiotics, thereby representing a possible new approach for the management of chronic biofilm related infections. In view of the potential of this enzyme cocktail to clear biofouling and black gunk from the drainage pipes (as reported earlier, Kaur et al. 2020a) and its presently assessed biomedical application, indicating clearance of biofilms from the biomedical devices, a patent has been filed (Indian patent, Application No. 202011003836 dated 28/01/2020).

## Supplementary Information

**Additional file 1: Figure S1.** Contour plots representing yields of different enzymes from the surface culture of *A. niger* APS on wheat bran and kitchen waste-based medium as affected by corn steep liquor (CSL; %) and MgSO_4_ (%) keeping the other variables at 0 coded values. **Figure S2.** (a) Well diffusion assay on a skimmed milk agar plate for the assessment of protease activity in the enzyme cocktail along with the positive controls, A: chymotrypsin (1.0 mg/ml), B: trypsin (1.0 mg/ml), C: proteinase K (1.0 mg/ml), D: enzyme supernatant, E: neutralized enzyme supernatant; (b) Well diffusion assay on glycerol tributyrate plate for the assessment of lipase activity in the enzyme cocktail along with the positive control (commercial lipase) at different dilutions; A: 2.0 mg/ml, B: 1.0 mg/ml, C: 0.5 mg/ml, D: 0.25 mg/ml, E: 0.125 mg/ml, F: Enzyme supernatant from *A. niger* APS.

## Data Availability

All data generated or analysed during this study are included in this article and its Additional files.
