# Supplementary material for: Cocktail of carbohydrases from Aspergillus niger: an economical and eco-friendly option for biofilm clearance from biopolymer surfaces
Source: AMB Express. 2021 Feb 4;11:22. doi: 10.1186/s13568-021-01183-y (PMC7862497; doi:10.1186/s13568-021-01183-y)
Supplement: Supplementary file 1 — Additional file 1: Figure S1. Contour plots representing yields of different enzymes from the surface culture of A. niger APS on wheat bran and kitchen waste-based medium as affected by corn steep liquor (CSL; %) and MgSO4 (%) keeping the other variables at 0 coded values. Figure S2. (a) Well diffusion assay on a skimmed milk agar plate for the assessment of protease activity in the enzyme cocktail along with the positive controls, A: chymotrypsin (1.0 mg/ml), B: trypsin (1.0 mg/ml), C: proteinase K (1.0 mg/ml), D: enzyme supernatant, E: neutralized enzyme supernatant; (b) Well diffusion assay on glycerol tributyrate plate for the assessment of lipase activity in the enzyme cocktail along with the positive control (commercial lipase) at different dilutions; A: 2.0 mg/ml, B: 1.0 mg/ml, C: 0.5 mg/ml, D: 0.25 mg/ml, E: 0.125 mg/ml, F: Enzyme supernatant from A. niger APS. [file 13568_2021_1183_MOESM1_ESM.docx]

**Journal: AMB Express**

**Cocktail of carbohydrases from *Aspergillus niger*: an economical and eco-friendly option for biofilm clearance from biopolymer surfaces**

Arashdeep Kaur^a^, Sanjeev Kumar Soni^a^, Shania Vij ^a^, Praveen Rishi^a^*****

^a^ Department of Microbiology, Panjab University, Chandigarh, 160014, India.

***Corresponding Author:**

Dr. (Mrs.) Praveen Rishi, Ph.D., FAMI, FABMS

Professor, Department of Microbiology

Panjab University

Sector- 25, Chandigarh-160014, India.

E mail: [rishipraveen@yahoo.com](mailto:rishipraveen@yahoo.com); [rishiparveen@pu.ac.in](mailto:rishiparveen@pu.ac.in)

Contact No. +919888895206

ORCID ID: 0000-0001-5425-8064

**
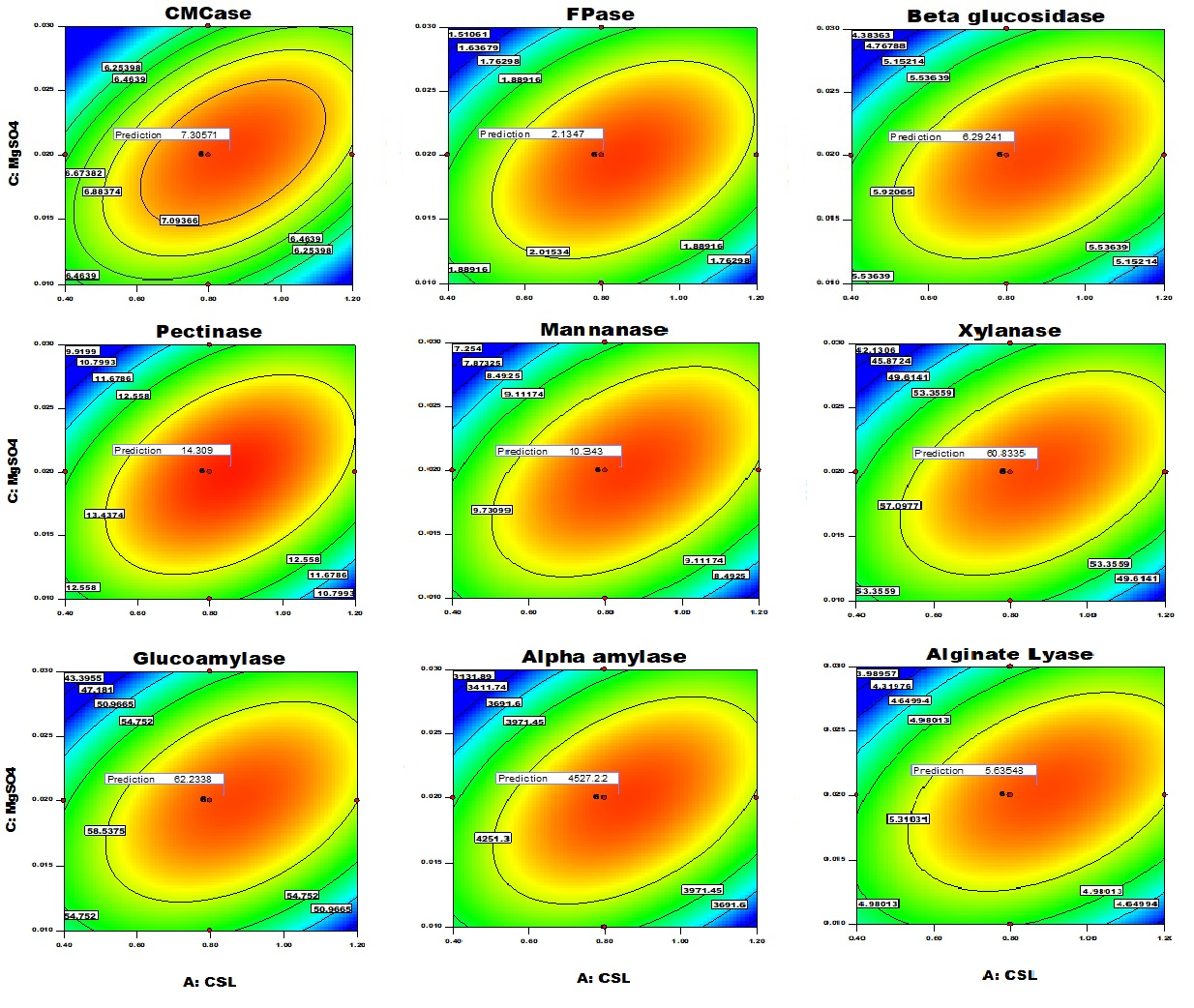
**

**Fig S1** Contour plots representing yields of different enzymes from the surface culture of *A. niger* APS on wheat bran and kitchen waste-based medium as affected by corn steep liquor (CSL; %) and MgSO_4_ (%) keeping the other variables at 0 coded values

**Fig. S2** (a) Well diffusion assay on a skimmed milk agar plate for the assessment of protease activity in the enzyme cocktail along with the positive controls, A: chymotrypsin (1.0 mg/ml), B: trypsin (1.0 mg/ml), C: proteinase K (1.0 mg/ml), D: enzyme supernatant, E: neutralized enzyme supernatant; (b) Well diffusion assay on glycerol tributyrate plate for the assessment of lipase activity in the enzyme cocktail along with the positive control (commercial lipase) at different dilutions; A: 2.0 mg/ml, B: 1.0 mg/ml, C: 0.5 mg/ml, D: 0.25 mg/ml, E: 0.125 mg/ml,F: Enzyme supernatant from *A. niger* APS
